# On the Common Journey of Neural Cells through Ischemic Brain Injury and Alzheimer’s Disease

**DOI:** 10.3390/ijms22189689

**Published:** 2021-09-07

**Authors:** Jan Kriska, Zuzana Hermanova, Tomas Knotek, Jana Tureckova, Miroslava Anderova

**Affiliations:** 1 Institute of Experimental Medicine, Czech Academy of Sciences, 142 20 Prague, Czech Republic; jan.kriska@iem.cas.cz (J.K.); zuzana.hermanova@iem.cas.cz (Z.H.); tomas.knotek@iem.cas.cz (T.K.); jana.tureckova@iem.cas.cz (J.T.); 2 Second Faculty of Medicine, Charles University, 150 06 Prague, Czech Republic

**Keywords:** central nervous system, ischemic brain injury, stroke, Alzheimer’s disease, dementia, neurodegeneration, amyloid beta, Wnt signaling

## Abstract

Ischemic brain injury and Alzheimer’s disease (AD) both lead to cell death in the central nervous system (CNS) and thus negatively affect particularly the elderly population. Due to the lack of a definitive cure for brain ischemia and AD, it is advisable to carefully study, compare, and contrast the mechanisms that trigger, and are involved in, both neuropathologies. A deeper understanding of these mechanisms may help ameliorate, or even prevent, the destructive effects of neurodegenerative disorders. In this review, we deal with ischemic damage and AD, with the main emphasis on the common properties of these CNS disorders. Importantly, we discuss the Wnt signaling pathway as a significant factor in the cell fate determination and cell survival in the diseased adult CNS. Finally, we summarize the interesting findings that may improve or complement the current sparse and insufficient treatments for brain ischemia and AD, and we delineate prospective directions in regenerative medicine.

## 1. Introduction

Neuropathologies such as cerebral ischemia and Alzheimer’s disease (AD), negatively influence the lives of a large number of people worldwide [1,2]. They both affect the correct functioning of the central nervous system (CNS), and recently a plethora of links between brain ischemia and AD have been identified. Among others, they also share several environmental risk factors, such as physiological aging and unhealthy lifestyle. Additionally, they are characterized by common pathological processes such as dysregulated expression of Alzheimer-related genes, neuroinflammation, reactive gliosis, and aberrant mitochondrial function, which all lead to neurodegeneration [3,4]. Therefore, it is crucial to study the processes that occur in both CNS pathologies to reveal potential new treatments for them. In this review, we focus on the pathophysiology of cerebral ischemia and AD, with their impact on CNS functioning, and summarize current knowledge about ischemic injury to improve understanding of the onset and the course of AD. Moreover, we introduce the Wnt signaling pathway, an important player in both ischemia and AD, as a possible field of interest in pursuit of new therapeutic targets to cure neurodegenerative diseases. Finally, we summarize the ongoing efforts that attempt to slow, stop, or even reverse the progression of these neuropathologies. Overall, we believe that a comprehensive investigation into cerebral ischemia may lead to the development of a treatment for AD patients, that overcomes the neurological problems that accompany the eventual loss of cells associated with the disease.

## 2. Ischemic Brain Injury

Cerebral ischemia is one of the leading causes of death and adult disability worldwide [5]. It manifests through a number of symptoms that differ based on the type and localization of the injury: from transient monocular visual loss, hearing loss, vertigo, and dizziness, to difficulty speaking and sensory or motor symptoms affecting the limbs and face [6]. Ischemic brain injury is caused by a reduction in the blood flow, typically due to atherosclerotic disease, head trauma, or cardiac arrest, which then results in oxygen and glucose deprivation of the brain tissue. The sudden loss of blood circulation in cerebral ischemic injury can be restricted to a small area of the brain (focal cerebral ischemia (FCI)), or it can affect the whole brain parenchyma (global cerebral ischemia (GCI)) [7]. Global cerebral ischemia is characterized by the blood-brain barrier (BBB) disruption, cellular swelling, neuronal death, and by numerous processes leading to overall changes in the extracellular matrix, cellular morphology and behavior [8]. On the contrary, focal cerebral ischemia starts with locally reduced blood flow and spreads through the adjacent brain regions. Based on the functional studies, two distinct areas in the injured nervous tissue were described: the ischemic core and the penumbra. In the ischemic core, the blood flow is severely reduced and necrotic cell death occurs due to the total breakdown of homeostasis [9,10]. This area is surrounded by structurally intact tissue with impaired functions, the penumbra [9,10,11]. The cells in the penumbra are unable to maintain their electrical activity; however, their energy supply is partially preserved, which is sufficient for their survival. This area can be spared if the blood flow, together with the oxygen supply, is fully restored; the permanent damage is then proportional to the duration of the blood circulation loss. Alternately, the ongoing chemical processes in the cells result in the blending of the penumbra with the ischemic core within the hours and days following the ischemic injury [9,10,12,13].

The insufficient blood flow in the penumbral area forces the glial cells, such as astrocytes, to undergo numerous changes and to become reactive [14]. Reactive astrocytes have a common feature of the high expression of glial fibrillary acidic protein (GFAP). In the penumbra, astrocytes that become reactive change their morphology and functions; their processes elongate and point towards the ischemic core and, in general, the cells become hypertrophic with an increased diameter and volume of highly branched processes [15]. Aside from the morphological changes, the functions of reactive astrocytes are also altered. Reactive astrocytes participate in the formation of the glial scar, which separates the ischemic core from the relatively healthy nervous tissue surrounding the ischemic lesion [16]. Moreover, reactive astrocytes participate in the removal of neuronal, and even immune cell debris from the damaged tissue. The ability to enwrap and engulf detrimental particles, also known as phagocytosis, is usually credited to microglia, the resident immune cells of the CNS; nevertheless, ischemia causes molecular changes in astrocytes that enable them to participate in these “cleaning processes”. However, it is worth mentioning that they act slower and with lower efficacy than microglia [17]. Like astrocytes, microglia reach an activated state in response to ischemic brain injury. In the early stages of ischemia, they display the so-called M2 phenotype; if this phenotype is also maintained in the later stages, microglia protect against neuronal damage. On the other hand, as the pathology progresses, M2 microglia turn into M1 microglia, and the latter have an opposing effect, contributing to neuronal damage and cell death [18,19,20].

Within the ischemic tissue, a set of chemical processes is triggered. This relay is called the ischemic cascade and it begins with energy depletion, due to oxygen and glucose deprivation. Since the energy production for the brain is highly dependent on oxidative phosphorylation and glycolysis, the decreased supplies of oxygen and glucose cause the acute failure of the adenosine triphosphate (ATP)-dependent transporting systems, and lead to the disruption of ion homeostasis. The ionic imbalance causes spreading depolarization in neurons and glial cells, which occurs within minutes following ischemic injury [10,11,12,21]. Furthermore, the insufficient supply of oxygen activates anaerobic glycolysis and leads to the accumulation of lactate in the ischemic nervous tissue and the cerebrospinal fluid (CSF), where it contributes to secondary neuronal damage [22]. At the same time, spreading depolarization causes the dysfunction of voltage-dependent calcium (Ca^2+^) channels, strongly increases the intracellular levels of Ca^2+^, and results in the release of glutamate and other excitatory amino acids into the extracellular space [7,12]. In addition, cerebral ischemia and subsequent reperfusion cause the profound generation of reactive oxygen species (ROS) in mitochondria, which is stressful for most cells, and it therefore contributes to the damage of the whole brain tissue [23]. Ischemic brain injury leads to massive cellular damage, resulting in the apoptotic and necrotic cell death of different cell types, and to the development of brain edema [24,25,26].

## 3. Alzheimer’s Disease

Alzheimer’s disease is a serious neurodegenerative disorder that primarily affects the memory and cognitive functions and is the cause of 60–80% of all dementia cases in the elderly [27]. Based on the recent meta-analyses, the prevalence of the disease is approximately 1–3% in the 65–74 age group, 7–17% aged 75–84 and 22–32% in people over 85 years of age, and this is expected to almost double every twenty years [28,29,30]. These numbers suggest that AD is a disease in which age plays a major role. Worldwide, the average prevalence of the disease is between 5% and 7%, but there are differences between countries and continents [31,32,33], which is mainly explained by different living standards, quality of health care, and overall life expectancy [34,35]. Since AD is a disease that mainly affects the elderly, its incidence correlates with the life expectancy of the population. As the life expectancy is higher for women than for men [36], this is a likely explanation for the slightly increased prevalence and incidence in women, compared to men [27,28]. Diagnosis consists of demonstrating the cognitive or behavioral symptoms, or functional decline, and excluding dementia from other causes. This is done by a combination of neuropsychological tests, laboratory tests, genetic analyses, and imaging methods, namely magnetic resonance imaging (MRI) and positron emission tomography (PET). Laboratory tests are used to determine AD-specific biomarkers, such as amyloid β (Aβ) and tau protein, in the CSF or plasma. The MRI analysis reveals specific cerebral atrophy, mainly in the hippocampus and the medial temporal lobe, and the enlargement of the ventricles [37,38]. The PET imaging, in combination with specific pharmaceutical tracers, provides the detection of characteristic protein aggregates [39].

Two forms of AD have been described based on inheritance: familial (FAD) and sporadic (SAD) form [40]. The very rare (less than 5%) FAD occurs in younger individuals and is associated with mutations in causal genes such as amyloid precursor protein (APP) and presenilins 1 and 2 (PSEN1, PSEN2). The SAD is more common and is caused by a set of different factors including genetic predispositions and environmental risk factors. The most important genetic factor is a member of the apolipoprotein E (ApoE) family, namely the *ApoEε4* allele. The role of ApoE in the AD pathology is related to the impaired clearance and oligomerization of Aβ, with its subsequent accumulation in the brain [41,42,43]. The non-genetic risk factors include metabolic and cardiovascular disorders, immune system dysfunction, infectious diseases, head injury, exposure to metals, or psychiatric disorders [44,45]. 

The formation of extracellular amyloid plaques and intraneuronal neurofibrillary tangles (NFT), is considered to be the primary feature of AD. These structures result from the abnormal aggregation of misfolded Aβ proteins, and the phosphorylated form of the tau protein. In the area of plaques, neurodegeneration, astrogliosis, and microglia activation occur. The Aβ protein is also deposited in the blood vessels, where it causes amyloid angiopathy [46]. To some extent, the overproduction of Aβ and tau may be successfully regulated by phagocytosis and the proteases activity, or by clearance via the perivascular circulation and the glymphatic system. The defective clearance is considered to be one of the main causes of SAD [47,48]. Recent studies indicate that some Alzheimer’s-related brain changes, including Aβ plaques and NFT, are present in healthy aged people who never develop any clinical symptoms; however, it seems that only when they occur simultaneously, and are supported by other risk factors, AD may manifest [49,50,51]. The phosphorylated tau directly affects the internal cell processes and is responsible for neuronal dysfunction and cell death. Tau phosphorylation can be triggered by Aβ binding to neuronal receptors, and by the activation of tau kinases that modify the protein at specific residues [52,53,54]. The Aβ protein is a universal ligand that can bind to various receptors in different cell types and it therefore activates the processes that ultimately lead to changes in the neuronal metabolism, mitochondrial and synaptic function, inflammation, and neuronal loss, which results in dementia. The synaptotoxic effect of Aβ is most likely activated in several different ways. It includes the partial block of postsynaptic glutamate N-methyl-D-aspartate receptors (NMDARs) due to the internalization or desensitization, resulting in weakened long-term potentiation (LTP) and facilitated long-term depression (LTD) [55,56,57]. Additionally, the activation of the α7 nicotinic acetylcholine receptors (α7-nAcChRs), metabotropic glutamate receptors (mGluRs), or perisynaptic NMDARs results in the LTD facilitation via calcineurin-STEP-cofilin, mitogen-activated serine/threonine protein kinase p38 MAPK (p38 MAPK), and glycogen synthase kinase 3β (GSK3β) signaling pathways [55,58,59]. Furthermore, some other receptors that are likely to interact directly with Aβ, and affect dendritic spine loss, synaptic dysfunction, and actin depolymerization, have been described (e.g., cellular prion protein, ephrin receptors, and p75 neurotrophin receptor) [60]. The Aβ protein is produced mainly, but not exclusively, in neurons from APP via the activity of β- and γ-secretase. Importantly, the product is a mixture of variants differing in their length, solubility, and biological and toxic properties. The most common variant in the healthy tissue and in the tissue affected by AD is Aβ40, while in AD the Aβ42 variant predominates. Recent results suggest that it is not amyloid plaques, but small soluble oligomeric structures such as Aβ oligomers that bind to the receptors in the cell membrane, and thus hinder cell signaling and other cellular functions [61,62,63]. Additionally, it has been shown that they affect synaptic plasticity [64] and cognitive functions [57], following the implantation into experimental animals, and these findings coincided with the onset of the symptoms in the AD model [65].

Although for several years research on AD has been mainly focused on the events leading directly to neurodegeneration, and has overlooked the function of other cell types, it is now clear that the pathology of AD is a complex process that not only includes changes in the distribution, function, and interactions among neurons, astrocytes, and microglia, but also other cell types, such as oligodendrocytes or monocytes.

In the healthy organism, microglia function as protective cells that contribute to the maintenance of the brain homeostasis. Due to their ability to phagocytose, they scavenge waste products, excess or dead cells, and infectious agents. They are also able to phagocytose synapses in a process called synaptic pruning, in which they remove dysfunctional synapses and thus take an active part in the remodeling of the synaptic circuits [66,67,68]. In pathology, microglia become reactive and they usually occur in two phenotypes, one produces pro-inflammatory factors while the other, the protective one, produces anti-inflammatory cytokines and neurotrophins. In AD, microglia are recruited to the site of neurodegeneration by molecules released by neurons (fractalkine), as well as by astrocytes (glia-derived neurotrophic factor) [69,70,71]. The recruited microglia become activated, and they transform into inflammatory mononuclear phagocytes; these then negatively affect the neuronal function via several different mechanisms from the phagocytosis of axonal projections, through complement-mediated lysis, to the cytokine-induced neuronal damage. In AD, microglia typically produce pro-inflammatory cytokines, such as tumor necrosis factor alpha (TNFα), interleukins (IL1β, IL1α, IL6), and interferon γ (IFN-γ), chemokines, growth factors, and nitric oxide (NO), a typical marker of inflammatory activation [72,73,74]. The production of IFN-γ, TNFα, and IL1β, in turn triggers astrocyte proliferation and activation [75,76]. In addition, microglia lose their ability to phagocytose Aβ with age, which also contributes to their impaired clearance from the brain tissue. Two genes, which are among the risk genes for AD, most likely play a role in the process of the uptake of Aβ by microglia. The triggering receptor expressed on myeloid cells 2 (TREM2), is a transmembrane receptor that has been shown to mediate microglial fagocytosis and activate the anti-inflammatory cytokine profile [77], while myeloid cell surface antigen CD33 regulates innate immunity [78].

Astrocytes are, under physiological conditions, homeostatic cells with a variety of protective, supportive, and nutritional functions. Via numerous processes, they contact and modulate neuronal synapses and form *glia limitans* of the BBB. In AD, astrocytes are, similar to microglia, attracted to the place of neurodegeneration by C–C motif chemokine ligand 2 (CCL2) and Aβ, produced not only by neurons and microglia, but also by activated astrocytes [69,70,79,80]. The attracted astrocytes become reactive, as their morphology and functions are altered. Morphological changes include the shortening of processes and the reduction of branching, which can result in impaired metabolic support and synaptic control. In return, reactive astrocytes produce increased amounts of chemokine ligands such as CCL3, CCL5, CXCL10, which attract and activate microglia. Astrocytes contribute to the overproduction of Aβ in several ways. Firstly, they produce exosomes that contain excessive amounts of APP and β-site APP-cleaving enzyme 1 (BACE1), which are then taken up by neurons, leading to the enormously increased Aβ42 production [81]. Secondly, reactive astrocytes themselves produce increased amounts of Aβ [76]. Additionally, since astrocytes participate in the Aβ clearance through their aquaporin 4 (AQP4) water channels, expressed on the processes surrounding cerebral vessels, changes in the AQP4 expression and polarization on astrocyte processes are likely to contribute to the impaired Aβ clearance [82,83,84]. Finally, the processes of astrocytic internalization and degradation of Aβ are malfunctioning, or at least limited, in AD [85]. One of the basic functions of astrocytes is to maintain glutamate homeostasis. Most extracellular glutamate is taken up by astrocytes via excitatory amino acid transporters (EAATs). Glutamate is then converted to glutamine, by glutamine synthetase (GS) and shuttled back into the neuronal presynaptic terminals in the process called the glutamate-glutamine shuttle. If this mechanism is impaired, neuronal NMDARs are overstimulated and synaptic dysfunction ensues. In AD, a decreased expression of both glutamate transporters, glutamate transporter-1 (GLT-1) and glutamate aspartate transporter (GLAST) has been observed in the immediate vicinity of amyloid plaques, which resulted in decreased glutamate clearance [86]. The roles of astrocytes in AD are summarized in more detail in [87] and [88]. Glial cells that are rather neglected in connection with AD, are the cells of the oligodendroglial lineage. Myelin loss has been reported in patients with AD, resulting in a progressive disconnection of neural networks [89]. An increased proliferation of oligodendrocyte precursor cells (OPCs) has been observed in a mouse model of AD, indicating the activation of the reparative processes. However, a similar reaction was not observed in AD patients [90]. Interestingly, OPCs can also phagocytose Aβ, and thus contribute to its clearance [91].

## 4. Common Features of Ischemic Brain Injury and Alzheimer’s Disease

Initially, it might appear that cerebral ischemic injury and AD are two completely different disorders of the CNS. However, the last decades of research have revealed that they not only begin at “similar predispositions”, but that they also share a common journey, with a final destination at “cell death”. Recently, new observations have been made, unveiling parallels in the pathophysiology of brain ischemia and AD, and their contribution to the accumulation of Aβ peptide and subsequent neuronal death. Moreover, growing evidence suggests that brain ischemia may be implicated in the etiology of AD [92].

Genetic and environmental predispositions represent a substrate for CNS disorders. Ischemic brain injury and AD share several common risk factors; however, they have little in common when it comes to genetic predispositions. About 70% of the risk of developing AD is attributed to genetics. Mutations associated with the disease are predominantly identified in the genes for APP, PSEN1, PSEN2, and ApoE [93]. On the other hand, recent studies have reported a long list of candidate risk alleles for stroke. The most promising genes were involved in neuroinflammation (tetraspanin 2), atherosclerosis (histone deacetylase 9), and lipid metabolism (ApoE, predominantly associated with hemorrhagic stroke); however, no risk alleles have been conclusively approved so far. This could be explained by the probable multifactorial nature of the majority of stroke cases, with combined genetic and environmental risk factors of minor influence [94,95]. Therefore, in our search for common onset factors of cerebral ischemia and AD, it is more advantageous to assess the environmental factors.

One of the factors that increases the susceptibility of the organism to diseases, and triggers the pathophysiology, is physiological aging. This risk factor dramatically reduces the lumen and increases the fibrosis of blood vessels [96], which, together with an age-dependent decrease of brain ischemic tolerance, predetermine older age as one of the risk factors for brain ischemia [97]. The age-dependent vascular changes are also a probable pathogenic contributor in age-related dementia such as AD [98]. The aging of the human brain as a reason for developing dementia can be substantiated by the fact that a normal brain weight is approximately 1300 g, while the average brain weight of 100-year-olds is under 1100 g [99]. Another recent finding comes from the research field of glial cells. Habib et al. [100] identified a population of disease-associated astrocytes in a murine model of AD, while similar cells were also found in aged wild-type mice, as well as in the aging human brain.

Another significant environmental risk factor in the development of cerebral ischemia and AD is an unhealthy lifestyle, such as an imbalanced, high-fat diet, poor physical activity, and smoking. For example, dietary and lifestyle interventions may mitigate the aging effects of ApoE4, an allele that elevates the risk of age-related disorders of the arteries and the brain. On top of that, this allele is associated with glucose dysregulation and body weight, which further increases the risk of neurodegeneration [101]. Furthermore, countless studies have been conducted to assess the relationship between smoking and stroke, and the data clearly show that smokers have an overall increased risk of stroke by ~92%, when compared to non-smokers. Importantly, even passive smoking increases the overall risk of stroke by ~45% [102]. Moreover, a higher AD prevalence among smokers has been identified, which may be explained by the fact that cigarette smoke components interact with Aβ, and thus promote its aggregation [103]. A great number of these environmental factors are modifiable and, to prevent the complications associated with cognitive decline or to improve quality of life, protective measures such as a healthy lifestyle should be taken [93].

In addition to the above-mentioned risk factors, brain ischemia and AD share several processes and mechanisms that lead to the loss of cells and the malfunctioning of the CNS. One of the findings that connect brain ischemia and AD is the dysregulated expression of Alzheimer-related genes upon ischemic injury, which makes stroke itself a risk factor for late-onset of SAD [104,105]. Recent research clearly indicates that post-ischemic brain injury is associated with the deposition of folding proteins such as Aβ and tau, and that cerebral ischemia may be the triggering event of AD [106]. Initial, focally localized ischemic episodes may spread to neighboring parts of the brain parenchyma, causing ischemic degenerative changes that eventually turn into post-ischemic dementia with the Alzheimer’s phenotype. A more profound accumulation of neurotoxic APP, and the repressed recovery of neuronal growth-promoting microtubule-associated protein 1B (MAP1B) following FCI in aged rats, further suggested that brain ischemia may play a prominent role in the etiology of AD [107,108]. Coherently, following GCI, APP is predominantly stored in the hippocampus, the region of the brain most affected by AD [109]. Similarly, a significant accumulation of Aβ peptide and ApoE was identified in a mouse model of cerebral hypoperfusion, as well as in ischemic patients, indicating a possible novel therapeutic strategy for AD in the prevention of ischemic insults [110,111]. Following GCI, an over-expression of APP caused a subsequent increase in the production of Aβ peptide, which in turn promoted vascular dysfunction and degenerative changes in the function of surviving neurons and the BBB, eventually leading to post-ischemic dementia with Alzheimer’s phenotype [112]. The ischemic BBB may trigger the accumulation of neurotoxic molecules such as phosphorylated tau protein, causing the formation of neurofibrillary tangles and thus interfering with the proper microtubule functions in neurons [113]. This has also been observed in the hippocampal CA1 region of the rat [114]. Additionally, the AD-associated proteins were not only identified in neurons, but also in glial cells. Upregulated Aβ peptide was detected during reactive astrogliosis in the glial scar [112]. More specifically, brain ischemia temporarily induced Aβ over-expression in the cytoplasm of reactive astrocytes, with peaks at seven days and six months after reperfusion [115]. Moreover, GCI is defined by delayed neuronal death in specific regions of the brain such as the CA1 region of the hippocampus, and by reactive gliosis [8,116]. Interestingly, the potential role of glial cells as targets for neuroprotection has been revealed, since it was disclosed that the apoptosis of astrocytes contributes to the pathogenesis of AD and several other degenerative disorders [117]. The role of glial cells in brain ischemia and AD, corroborates another shared feature of the two CNS disorders. Neuroinflammation and reactive gliosis are both associated with Aβ peptide storage in AD [115]. Similarly, glutamate-related excitotoxicity, together with high levels of TNFα, are responsible for inflammatory stimulation in brain ischemia [118], and together with glial activation and proliferation [8,119], these aspects resemble those found in AD. The progression of AD and brain ischemia are both characterized by the activation of glial cells and the upregulation of neuroinflammatory signals [120], which may even persist two years after ischemia-reperfusion brain injury [121]. Additionally, NG2 glia, an abundant population of glial cells in the adult mammalian CNS, are capable of generating reactive astrocytes in the lesioned brain [122]. Nevertheless, NG2 glia proliferate and only transiently differentiate to astrocytes upon severe insults to the CNS, such as brain ischemia [123,124,125], while only a negligible fraction of NG2 glia expresses GFAP, a marker of reactive astrocytes, in chronic types of CNS injuries such as AD [124]. Similarly to cerebral ischemia, not only gray but also white matter of the adult brain is affected by AD. Amino acid glutamate is necessary for oligodendrocyte differentiation and maturation; nevertheless, aberrantly elevated levels of this neurotransmitter under ischemic conditions lead to intracellular Ca^2+^ accumulation and mitochondrial dysfunction. This resulted in white matter injury in rats and mice [126]. Despite the belief that AD is only responsible for pathological changes in gray matter, degenerative abnormalities in white matter were also identified. The observed degeneration of oligodendrocytes, compensated by a higher incidence of NG2 glia (also known as OPCs), was associated with markedly decreased amounts of myelin basic protein, myelin proteolipid protein, and cyclic nucleotide phosphohydrolase, all indicating demyelination [127]. Moreover, the crosstalk between the dysfunctional mitochondria and the endoplasmic reticulum is disturbed in cerebral ischemia and AD, interfering with proper energy metabolism, and Ca^2+^ and lipid homeostasis, and increasing inflammation and autophagy [128]. The selective degradation of damaged mitochondria by autophagy is called mitophagy. This mechanism is vital for maintaining mitochondria homeostasis and has been related to ischemic injury [129,130] as well as neurodegenerative diseases including AD [131]. Interestingly, it has been disclosed that mitophagy inhibits Aβ and tau pathology, reversing memory impairment and preventing cognitive deficits in animal models of AD [132]. Therefore, mitophagy may represent a viable therapeutic intervention.

The ability of astrocytes to respond to pathological stimuli, together with the function of glutamate transporters, is compromised in the aged hippocampus [133]. As stated above, ischemia triggers glutamate excitotoxicity that is characterized by a cell membrane depolarization, Ca^2+^ overload, and extracellular accumulation of glutamate. These processes are accompanied by the formation of free radicals, oxidative stress, edema, inflammation, and the loss of synapses [7], and may cause cell death during various brain pathologies [134]. In AD, synaptic glutamate NMDARs promote cell survival, while extrasynaptic NMDARs initiate cell death and thus contribute to the etiology of the disease [135]. Moreover, following the accumulation of abnormally folded Aβ protein, an early synaptic dysfunction was observed [136]. Additionally, Kulijewicz-Nawrot et al. [137] disclosed a reduced expression of GS, an astrocyte-specific enzyme that converts glutamate to glutamine, in a triple-transgenic mouse model of AD (3xTg-AD). This decrease compromises glutamate homoeostasis, which may result in failures in synaptic connectivity, leading to deficient cognition and memory. Therefore, the use of NMDARs antagonists such as memantine or dizocilpine (also known as MK801), might represent a new treatment for AD [86,138].

Another common feature of cerebral ischemia and AD is that they afflict specific CNS structures. A cell type highly susceptible to global ischemic injury is the CA1 pyramidal neuron of the hippocampus [139]. This region is also preferentially related to brain atrophy and memory deficits observed in the elderly diagnosed with AD [140]. Besides the hippocampus, another brain structure that undergoes harmful age-related changes is the BBB. This structure forms a physiological interface between the brain parenchyma and the vasculature. In health, it effectively mediates the exchange of vital substances in the CNS, while preventing the influx of detrimental solutes [141]. In cerebral ischemia, its disruption and subsequent increased permeability are the critical pathological processes compromising normal neuronal functions. As a result, dysregulated water and ion homeostasis leads to cerebral edema, and infiltrating leukocytes aggravate inflammatory responses commenced by residing microglia [142,143]. Additionally, it has been proposed that together with the selective neurodegeneration of vulnerable neurons, chronic changes in the BBB may accelerate the progression of the ischemic brain tissue pathology leading to post-ischemic dementia [112]. Moreover, traumatic brain injury (TBI) has been identified as a predisposing risk factor for AD and cerebral ischemia [144]. Alterations in the BBB permeability after TBI lead to the invasion of immune cells and, more importantly, to the accumulation of aggregation-prone molecules that are implicated in the progression of neurodegenerative diseases with dementia [145]. Transient receptor potential cation channel subfamily V member 4 (TRPV4), was found to be one of the therapeutic targets for TBI [146]. Importantly, water channel AQP4 and ion channel TRPV4, are expressed on astrocyte endfeet and regulate water and ion fluxes through the walls of blood vessels [147,148]. Interestingly, an increased expression of both channels in astrocytes following cerebral ischemia was observed [149,150], while alterations in their distribution were also confirmed during aging and AD progression [151,152]. Therefore, a better understanding of the pathophysiological roles of the AQP4 and TRPV4 channels is vital for pursuing new therapeutic strategies.

**Figure 1:**
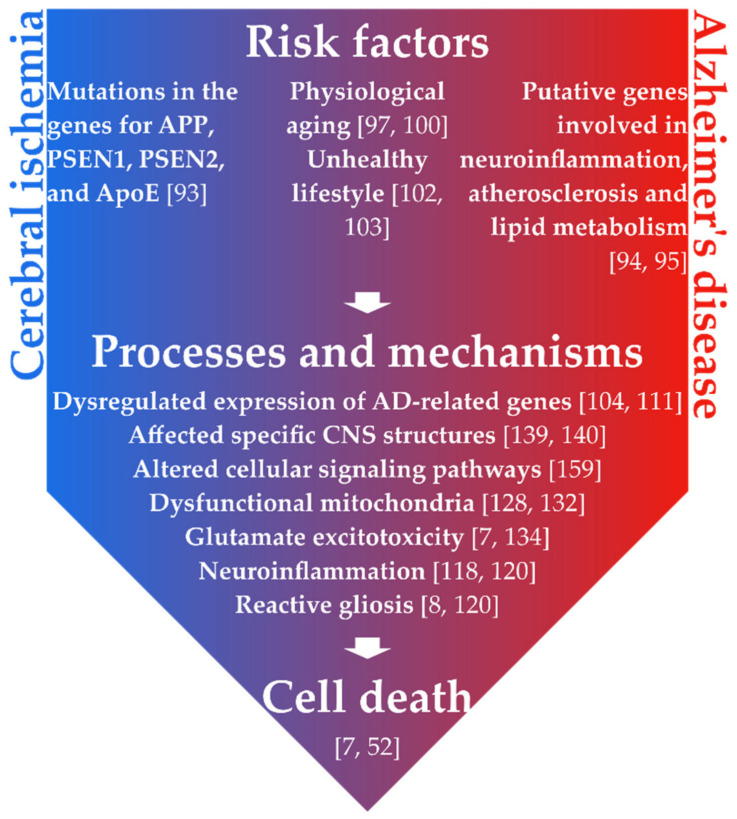
Common journey of neural cells through ischemic brain injury and Alzheimer’s disease. Despite diverse genetic predispositions, cerebral ischemia and Alzheimer’s disease (AD) share common risk factors and pathophysiological processes and mechanisms, all leading to cell death. For more information, please refer to the main text and the publications indicated by the reference numbers in square brackets. Abbreviations: ApoE, apolipoprotein E; APP, amyloid precursor protein; CNS, central nervous system; PSEN1/2, presenilin 1/2.

To emphasize, it is crucial to identify where brain ischemia and AD go their separate ways and where their paths cross. Indeed, these reunions of the two runaways from the normal, physiological functioning of the CNS are of great importance, since these “crossroads” (Figure 1) may help us decipher what is important and what might be useful for the prospective therapeutic purposes. Moreover, all cellular processes are largely dependent on intrinsic and extrinsic molecular signals, such as growth factors or various components of cellular signaling pathways. On top of that, it is important to realize that these molecular inputs regulate cellular processes not only in health, but also during the pathological states of the CNS [125,153]. There are several cellular signaling pathways that may represent such “crossroads”, connecting brain ischemia and AD; recent research has indicated candidates such as Notch, signal transducer and activator of transcription 3 (Stat3), or Wnt signaling. Numerous studies have linked mutations in Notch 3 receptor to progressive disorder cerebral autosomal dominant arteriopathy with subcortical infarcts and leukoencephalopathy, the most common form of hereditary stroke. Notch signaling is also implicated in learning, synaptic plasticity and neurogenesis, as well as in the modulation of APP and the production of Aβ in neurons, all processes dysregulated in the progression of AD [154,155]. Stat3, on the other hand, has been associated with reactive astrogliosis in stroke and AD. The upregulation of this pathway has been identified at the border of the infarction zone and its conditional deletion, specifically in reactive astrocytes, was neuroprotective three days after transient middle cerebral artery occlusion (MCAO) [156]. Interestingly, Stat3 was also activated in reactive astrocytes around the plaques in AD. The inhibition of Stat3-mediated astrogliosis reduced Aβ levels and plaque burden, while it increased microglial amyloid phagocytosis, which resulted in ameliorated spatial learning and memory decline in a mouse model of AD [157]. Importantly, another cellular signaling, the Wnt pathway, is heavily influenced by aging [158,159], the main risk factor of both brain ischemia and AD. Moreover, Wnt signaling is also implicated in the processes such as glutamate signaling and synapse modulation, while active Wnt signaling positively regulates cell survival, and also the proliferation and differentiation of neural cells in humans [159,160]. Our previously published results, together with unpublished data, also suggest that *Wnt5a*, *Wnt7a*, and *Wnt7b* ligands and several downstream components of Wnt signaling, such as low-density lipoprotein receptor-related protein 5/6 (*Lrp5/6*), axis inhibition (*Axin2*), T-cell factor 7-like 2 (*Tcf7l2*), and tumor necrosis factor receptor superfamily, member 19 (*Troy*), play an important role in physiology and pathology of the CNS and in the cell fate specification [123,124,161,162]. Therefore, from our point of view, Wnt signaling acts as the common denominator of the two neuropathologies. Additionally, the Wnt pathway has been found to be active in the cells residing in the hippocampus, the region that is compromised in cerebral ischemia and AD. For these reasons, we further direct our attention to Wnt signaling, and discuss its functions in the two CNS disorders.

## 5. Wnt Signaling in Ischemic Brain Injury and Alzheimer’s Disease

**Figure 2:**
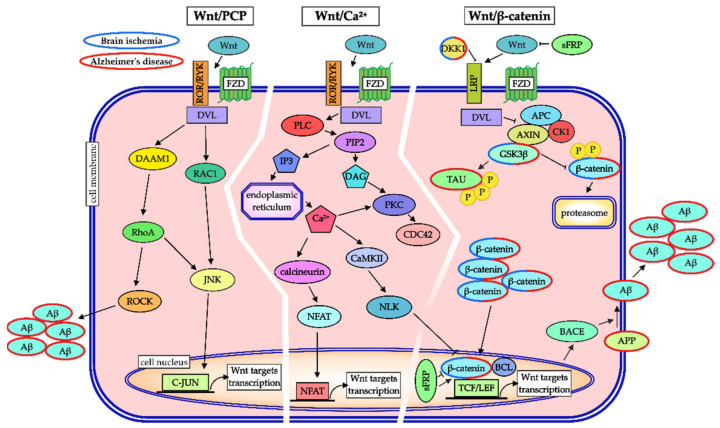
Participation of Wnt signaling in ischemic brain injury and Alzheimer’s disease. The scheme depicts three Wnt signaling pathways: two non-canonical branches, the planar cell polarity (Wnt/PCP) pathway and the Wnt/calcium (Ca^2+^) pathway, and the canonical, Wnt/β-catenin pathway. Wnt signaling is implicated in several processes associated with brain ischemia (indicated by blue outlines of the proteins) and Alzheimer’s disease (indicated by red outlines of the proteins). For more information, please refer to the main text. Abbreviations: Aβ, amyloid β; APC, adenomatous polyposis coli; APP, amyloid precursor protein; AXIN, axis inhibition; BACE, β-site APP-cleaving enzyme 1; BCL, B-cell lymphoma; C-JUN, transcription factor C-JUN; CaMKII, Ca^2+^/calmodulin-dependent protein kinase II; CDC42, GTPase CDC42; CK1, casein kinase 1; DAAM1, DVL-associated activator of morphogenesis 1; DAG, diacylglycerol; DKK1, dickkopf 1; DVL, disheveled; FZD, frizzled receptor; GSK3β, glycogen synthase kinase 3β; IP3, inositol trisphosphate; JNK, c-Jun N-terminal kinase; LRP, low-density lipoprotein receptor-related protein receptor; NFAT, nuclear factor of activated T-cells; NLK, nemo-like kinase; P, phosphorylation; PIP2, phosphatidylinositol 4,5-bisphosphate; PKC, protein kinase C; PLC, phospholipase C; RAC1, Rac family small GTPase 1; RhoA, small GTPase Ras homolog family member A; ROCK, Rho-associated kinase; ROR/RYK, tyrosine kinase receptors RYK/ROR; sFRP, secreted frizzled-related protein; TCF/LEF, transcription factors T-cell factor/lymphoid enhancer-binding factor; Wnt, Wnt protein/ligand.

In the adult CNS, Wnt signaling has been identified as an important factor affecting the cell fate and survival of neural cells [161,163]. Wnt proteins are members of a group of cysteine-rich secreted proteins that modulate the intercellular communication among cells in the vicinity, in an autocrine or paracrine manner [164]. They activate different cascades of Wnt signaling [165]; so far, three well characterized Wnt signaling pathways have been described the canonical Wnt pathway and two non-canonical branches of Wnt signaling: the planar cell polarity (PCP) pathway and the Wnt/Ca^2+^ pathway (Figure 2; [166,167]).

In the canonical Wnt pathway, the signal is relayed via β-catenin, the principal component of this Wnt branch. The signaling is initiated by the binding of a Wnt ligand to the extracellular domains of the frizzled (FZD) receptor and the LRP5/6 receptors, which attracts intracellular proteins disheveled (DVL) and AXIN to the cellular membrane [168]. These events prevent the formation of the so-called destruction complex, and result in β-catenin stabilization, with its subsequent translocation to the nucleus. In the nucleus, β-catenin binds to the transcription factors TCF/lymphoid enhancer-binding factor (LEF), which activates the transcription of Wnt-responsive genes. On the other hand, in the absence of the Wnt signal, the destruction complex consists of adenomatous polyposis coli (APC), AXIN, and two protein kinases: GSK3β and casein kinase 1 (CK1). The kinases are responsible for β-catenin phosphorylation and subsequent ubiquitination, leading to the degradation of β-catenin in the proteasome [169].

The non-canonical Wnt/PCP pathway consists of the FZD receptor and tyrosine kinase receptors of the RYK and ROR families [170]. Similarly to the canonical pathway, the signal from receptors is transduced to DVL; however, instead of β-catenin, the signal is carried by the small GTPase Ras homolog family member A (RhoA) or the Rac family small GTPase 1 (RAC1). One branch of this pathway relays the signal via DVL-associated activator of morphogenesis 1 (DAAM1). This protein is necessary for RhoA activation, which, together with Rho-associated kinase (ROCK), mediates actin polymerization. The other branch of the Wnt/PCP pathway is mediated by RAC1 which activates c-Jun N-terminal kinase (JNK). This kinase is responsible for the rearrangement of the cytoskeleton and for the phosphorylation of the C-JUN transcription factor, affecting cell proliferation, differentiation, and apoptosis [171,172].

In the Wnt/Ca^2+^ pathway, the signal is transmitted from DVL to phospholipase C (PLC) which is responsible for the hydrolyzation of phosphatidylinositol 4,5-bisphosphate (PIP2) to inositol trisphosphate (IP3) and diacylglycerol (DAG). Inositol trisphosphate promotes the release of intracellular Ca^2+^ from the endoplasmic reticulum. The elevated concentrations of Ca^2+^ together with DAG lead to the activation of protein kinase C (PKC), which cooperates with another GTPase, CDC42, and causes the rearrangement of the cytoskeleton. The release of Ca^2+^ is also responsible for the activation of calcineurin and Ca^2+^/calmodulin-dependent protein kinase II (CaMKII). Calcineurin interacts with the nuclear factor of activated T-cells (NFAT) transcription factor, regulating cell proliferation and differentiation, while CaMKII leads to the inhibition of the canonical branch of Wnt signaling via nemo-like kinase (NLK). For a more detailed description of the three Wnt signaling pathways, please, refer to [173,174], and [175].

Importantly, the activity of Wnt signaling can be modulated at several subcellular levels. For example, secreted frizzled-related proteins (sFRPs) act as biphasic modulators of Wnt signaling. On the one hand, they function as negative modulators of the pathway in the extracellular space and on the other hand, they affect the signaling in two opposing ways intracellularly. The nuclear form of sFRPs interacts with β-catenin and either promotes or suppresses Wnt signaling, depending on the binding site of the protein. Binding to the N-terminus of β-catenin represses the recruitment of TCF4 and thus inhibits Wnt signaling, while binding to the C-terminus of β-catenin upregulates the pathway [176]. Moreover, another extracellular protein, dickkopf 1 (Dkk1), antagonizes Wnt signaling by binding to the LRP co-receptor, which reduces the availability of the co-receptor to Wnt ligands [177,178]. Additionally, lithium chloride (LiCl) acts as an activator of the Wnt signaling pathway. Its mechanism consists in the inhibitory effect on the activity of a negative Wnt pathway regulator GSK3β. This rescue mechanism increases the abundance of cytoplasmic β-catenin, which leads to its translocation to the cell nucleus and hyper-activation of the pathway [179].

Recently, Wnt signaling has been associated with a plethora of neurological dysfunctions, including cerebral ischemia and AD [180,181,182,183,184]. Cerebral ischemia stems from a reduced cerebral blood flow and causes glutamate excitotoxicity, followed by neuronal death [7]. The only available means of treating brain ischemia is rapid vessel recanalization by the mechanical or pharmaceutical removal of the thrombus [185]. However, the beneficial roles of active Wnt signaling, especially its neuroprotective effects and stimulation of neurogenesis, were observed in ischemic brain injury [173].

It has been shown that Dkk1, a blocker of the canonical Wnt signaling pathway, contributes to the pathophysiology of neuronal death in a rat model of FCI. The Dkk1 protein was abundant in neurons of the ischemic core and the penumbra, following transient as well as permanent FCI. Moreover, the higher expression of Dkk1 protein was accompanied by reduced levels of β-catenin, while the treatment with lithium ions, which activate Wnt signaling, reversed this phenotype and showed a protective effect. These results indicate that the canonical Wnt pathway may play an important role in neuroprotection in stroke [186]; this effect can be explained by an indirect positive regulation of the anti-apoptotic protein B-cell lymphoma 2 (Bcl2) by β-catenin [187]. Importantly, higher plasma levels of Dkk1 have also been confirmed in patients with acute ischemic stroke, providing evidence that Wnt signaling is also implicated in the etiology of cerebral ischemia in humans. Interestingly, no correlation was identified between Dkk1 levels and the severity of the stroke [188,189]. The protective effect of the canonical Wnt pathway was also revealed in endothelial cells, where the treatment with LiCl or exogenous Wnt3a protein significantly decreased apoptosis induced by oxygen-glucose deprivation (OGD) [190]. Wnt signaling exerts its neuroprotective effect by blocking the activity of caspase 3 and thus preventing the apoptosis, not only in ischemia but also in AD [191]. Conversely, downregulated Wnt signaling led to more severe stroke and motor deficits that stemmed from mitochondrial dysfunction and neuroinflammation, developed under ischemic conditions. Nonetheless, these deleterious processes were reverted by the pharmacological inhibition of GSK3β; this activation of Wnt signaling also activated microglial autophagy after ischemic brain injury [192,193].

It is worth mentioning that high levels of estrogen in females act as an endogenous neuroprotective agent in stroke [194]. The mechanism behind this effect was revealed in hippocampal neurons, as it dwells in preventing the post-ischemic elevation of Dkk1 and, at the same time, activates canonical Wnt signaling [195]. Coherently with these findings, estrogen in ischemic animals subjected to GCI resulted in decreased levels of Dkk1, which correlated with increased levels of Wnt3a and an active nuclear form of β-catenin. In addition, it was demonstrated that even low physiological levels of estrogen protect the hippocampal CA1 region against GCI, while they also attenuate tau hyperphosphorylation, one of the hallmarks of the AD pathology [196]. These findings might have the power to unearth promising future implications of hormone therapy.

Ischemic stroke has been demonstrated to increase the proliferation potential of neural stem and progenitor cells (NSPCs) in the two neurogenic regions of the adult mammalian brain: the dentate gyrus (DG) of the hippocampus and the subventricular zone (SVZ) of the lateral ventricles (LVs). Adult hippocampal neurogenesis was also observed in humans [197]. Under ischemic conditions, a higher activity of Wnt signaling coincides with an increased proliferation and reduced apoptosis of NSPCs, which results in higher numbers of newly derived neuroblasts [198,199]. Moreover, Piccin and Morshead [200] disclosed upregulated Wnt signaling in response to stroke. This upregulation led to the switch from the asymmetric to the symmetric division of neural stem cells (NSC), while blocking the Wnt pathway inhibited their expansion. These observations suggest a mechanism by which Wnt signaling regulates the pool of NSC. A deeper investigation into this mechanism also revealed an increased expression of hypoxia-inducible factor 1α (HIF1α); either the knockdown of this factor or Wnt signaling inhibition counter-acted the hypoxia-induced proliferation of NSC [201]. In even more detail, ischemic injury/hypoxia stimulates the production of peroxynitrite, which in turn enhances the proliferation as well as neuronal differentiation of NSPCs via the activation of HIF1α and Wnt signaling [202]. Consistent with these findings, Zhang et al. [203] showed a significantly decreased differentiation of NSPCs to neuronal cells caused by the downregulation in the β-catenin expression. Concomitantly, in our study, we observed Wnt-signaling-driven differentiation of ischemia-activated adult NSPCs to neuroblasts. More specifically, we detected fewer neuronal precursors differentiated from NSPCs in vitro after Wnt signaling inhibition, while Wnt signaling hyper-activation increased the numbers of proliferating cells and neuroblasts in the SVZ in vivo [161]. Importantly, a decisive role of Wnt signaling in the promotion of adult neurogenesis, leading to enhanced functional recovery in a mouse model of FCI, was observed by Shruster et al. [204]. The above-mentioned observations in NSPCs may seem to contrast with the previously described notion about the increased expression of Dkk1 protein in stroke; however, Wnt signaling may affect terminally differentiated neurons and dividing NSPCs in the neurogenic niches differently, and an insult in the form of ischemic injury may be sufficient to awaken the dormant or quiescent multipotent cells residing in the adult brain.

Several studies disclosed that the activity of the canonical Wnt signaling pathway is required for postnatal vascular formation and maintaining the BBB integrity [205,206]. In the postnatal CNS, β-catenin signaling supports angiogenesis. This was documented by its depletion in genetically modified mice, which resulted in hypo-vascularization due to the deficient proliferation and sprouting of endothelial cells [207]. Consistent with the previous finding, an impaired and delayed vascularization in the retina of transgenic mice with inhibited canonical Wnt signaling was observed by Peghaire et al. [208]. Moreover, Wnt signaling has been identified as a link between brain angiogenesis and BBB formation, since the proper transition from vascular formation to BBB maturation is regulated by the temporal activity of this signaling [209]. Furthermore, Wnt/β-catenin signaling has been shown to be critical for promoting the expression of the BBB-specific glucose transporter 1 (GLUT1) and the tight junction protein claudin 5 [207], and together with its downstream target sex-determining region Y-box 17 (Sox17), it influences the maintenance and the permeability of the BBB [210]. Thus, these data suggest an indisputable function of Wnt signaling in angiogenesis and BBB maintenance, which might be helpful in the approaches trying to alleviate the damaging impacts of cerebral ischemia.

Besides ischemic stroke, the Wnt signaling pathway has also been identified as an important factor in AD. This signaling affects multiple aspects of the disease [211]. Similar to brain ischemia, alterations and dysregulations of Wnt signaling have been observed in both animal models of AD as well as in patients. Lower levels of proteins associated with active Wnt signaling were observed in the hippocampus of a mouse model of AD [212]. In accordance with this, changes in the activity of the Wnt signaling pathway components were observed in the prefrontal cortex of AD patients [213], and significantly higher levels of active GSK3β were detected in the neurons derived from AD patients [214]. Exceeding that, the proteins that are associated with the development of AD, namely PSEN1 and ApoE, were shown to interact with the Wnt signaling pathway components [215,216]. Similarly to ischemia, a high expression of Wnt signaling inhibitor Dkk1 was observed in autoptic samples of AD patients, also further indicating the importance of the Wnt signaling pathway in humans [217]. Thus, we believe that the dysregulation of Wnt signaling in AD is of utter importance and should be studied in more detail. Our belief may be seconded by the observation that the pivotal Wnt signaling kinase, GSK3β, has been associated with the production of Aβ and tau hyperphosphorylation. An increased production of Aβ42 and elevated levels of Aβ oligomers were observed after the inhibition of Wnt signaling, while its activation led to reduced levels of the Aβ42 aggregates [218]. Furthermore, a GSK3β-specific inhibition led to the reduced expression of β-secretase, which suppressed the cleavage activity of APP, and thus reduced the Aβ production. The decreased deposition of Aβ reduced plaque formation, and rescued memory deficits were observed after GSK3β inhibition in double transgenic AD mice [219]. Inhibitors of GSK3β, such as lithium or valproic acid, caused the decreased production of Aβ in vitro as well as in mice [220]. Interestingly, the effect of lithium probably depends on the progression of AD, since the treatment with this element promoted neurogenesis in two-month-old AD mice, but exerted no effect in six-month-old animals [221]. Additionally, the inhibition of the GSK3β activity decreased the expression of BACE1, which led to a reduced cleavage rate of APP and a lower production of Aβ [219]. It is worth mentioning that the crucial role of Wnt signaling in AD was also disclosed in the preclinical stage of the disease [222], which might pave the way to early detection and treatment.

The involvement of the Wnt pathway and GSK3β kinase was also documented in tau hyperphosphorylation. In neurons derived from AD patients, a higher activity of GSK3β was observed, which was associated with the elevated levels of phosphorylated tau [214]. In accordance with this observation, the loss of GSK3β inhibition was observed in aged transgenic mice and coincided with increased tau phosphorylation. Moreover, the pharmacological inhibition of GSK3β antagonized the age-dependent increase in tau phosphorylation [223]. Analogously, a decreased phosphorylation of virally introduced human TAU protein was detected in hemi-knockouts in the *Gsk3b* gene, when compared to wild-type mice. In these animals, the spread of TAU among neurons was also decreased. This suggests that the decrease in the GSK3β activity represses the induction of neurodegenerative changes exerted by tau, and that GSK3β might be a potential therapeutic target [224].

Importantly, the interactions between the Wnt pathways and the AD pathology are not merely unidirectional but they rather form a cycle where Wnt signaling affects the production of Aβ, while Aβ, in turn, influences Wnt signaling which results in a feedback loop [225,226]. One such mechanism was observed in cultured neurons that were exposed to Aβ. This caused a higher expression of Dkk1, while the knockdown of Dkk1 decreased the tau phosphorylation, together with the neuronal apoptosis induced by Aβ [217]. Tau also probably contributes to this loop, since the ablation of this protein prevented the activation of GSK3β by Aβ and, as a result, abolished the detrimental effect of Aβ on the axonal transport [227]. Alternatively, Aβ potentiated the tau hyperphosphorylation by GSK3β via a norepinephrine-dependent mechanism. The changes in norepinephrine signaling resulted in a huge 50- to 100-fold higher sensitivity of GSK3β to Aβ [53]. Overall, emerging evidence shows that Wnt signaling, Aβ, and tau protein are intertwined in a set of feedback regulations.

Nevertheless, the role of GSK3β is not only limited to the regulation of Aβ production, but it also plays a role in the neurotoxicity of amyloid plaques. Adding Aβ oligomers to neuronal cultures increased the activity of GSK3β and, at the same time, promoted apoptosis. However, the treatment with GSK3β inhibitors prevented Aβ-induced cell death [228]. The involvement of the Wnt signaling pathway was further demonstrated by the observation, showing that the Aβ synaptotoxicity is Dkk1-dependent. On top of that, Aβ synaptotoxicity was also increased by the activity of the non-canonical Wnt/PCP pathway, mediated by its branch regulating actin cytoskeletal dynamics via the DAAM1 protein [229]. In agreement with this, the blockage of Dkk1 by neutralizing antibodies led to suppression of Aβ-induced synaptic loss in mouse brain slices [230], and the downregulation of the Wnt/PCP downstream targets had a protective effect against the Aβ neurotoxicity [231]. However, it should be noted that the Dkk1-driven induction of the Wnt/PCP pathway only regulated Aβ toxicity in AD but failed to induce tau phosphorylation [231]. Therefore, it seems that Wnt signaling might also be involved in the neurotoxicity induced by Aβ plaques as a negative regulator.

Wnt signaling has been associated with memory improvement in AD mice and exhibited neuroprotective effects. To support these observations, the pharmacological inhibition of the GSK3β activity had a beneficial effect on neurogenesis, learning, and memory [232], and led to the decreased astrogliosis and microgliosis in animal models of AD [233]. The inhibition of Wnt signaling, on the other hand, resulted in cognitive deficits, increased tau phosphorylation, and elevated Aβ levels, which subsequently caused the abundance of larger senile plaques. Interestingly, the Wnt signaling inhibition also evoked similar pathological changes in wild-type mice [222]. Furthermore, the GSK3β inhibition led not only to reduced levels of phosphorylated tau in AD mice, but also to improved memory and slower progression of the disease [234]. Additionally, the inhibition of GSK3β by LiCl, or by genetic manipulation, led to an improved performance in the water maze, helped preserve the dendritic structure in the frontal cortex and the hippocampus, and decreased tau and APP phosphorylation, which also resulted in a decreased Aβ production [235]. This corroborates the findings in a model of early-onset AD showing that the deficiencies of presynaptic functions and the length of synapses were restored by Wnt signaling activators [236], while in vivo activation of the pathway improved synaptic functions and episodic memory, and enhanced LTP [237]. Similar effects were observed after the treatment with selenomethionine. This compound reduced Aβ deposition and decreased tau hyperphosphorylation, while improving the cognitive functions in 3xTg-AD mice. Selenomethionine has been linked to Wnt signaling; more specifically, it inhibited GSK3β and activated β-catenin/cyclin-D signaling, which promoted NSC proliferation. This led to increased numbers of hippocampal neurons; however, it also resulted in decreased counts of astrocytes [238]. Neurogenesis is a process that is orchestrated by the Wnt signaling pathway. The activation of Wnt signaling was observed in chronic hypoxia and, interestingly, this condition also led to an increased neurogenesis in double transgenic AD mice, suggesting a possible, and perhaps slightly unorthodox, therapeutic potential of mild hypoxia in the treatment of AD [239]. Additionally, the activation of canonical Wnt signaling inhibited the production of Aβ protein, and reduced the apoptosis in hippocampal neurons of 3xTg-AD mice. This observation indicates another possible way of utilizing the Wnt signaling pathway and its beneficial effects in the AD therapy [240].

Wnt/β-catenin signaling regulates the BBB formation, integrity, and function [178]. Simultaneously, the BBB breakdown is an early biomarker of human cognitive impairments in AD and has been associated with abnormal Wnt signaling. Importantly, ApoE, a major genetic risk factor for AD, promotes the degeneration of endothelial cells and hampers the clearance of Aβ exerted by pericytes. Moreover, brain endothelial cells connected by tight junctions and their main constituents, claudins, are Wnt/β-catenin target genes [241]. In line with this, Wnt7a and β-catenin knockouts in adult mice decreased the expression of claudins, which was accompanied by inflammation, insults to the CNS vasculature, and the dysregulated BBB maintenance. The loss of pericytes in human AD was also markedly higher in ApoE4 carriers, and it was associated with the BBB dysfunction [242]. Additionally, a decreased expression of GLUT1 at the BBB has been linked with the elevated Aβ accumulation and memory deficits. Interestingly, the Wnt pathway was associated with the correct functioning of the BBB-specific GLUT1 and glucose metabolism in an AD mouse model [243]. Specifically, the Wnt-induced activation of glucose metabolism resulted in an improved cognitive performance, while the downregulation of glucose uptake partially inhibited the beneficial effects of active Wnt signaling on learning and memory [244]. These findings indicate that the malfunctioning of Wnt signaling may contribute to a glucose metabolism deficiency in AD, revealing its potential therapeutic use [226].

Overall, recent research has suggested a common journey of neural cells through ischemic brain injury and AD. Physiological aging has been identified as a starting point with the strongest impact, while an unhealthy lifestyle is a modifiable environmental factor that can be prevented. Moreover, brain ischemia and AD are characterized by several common processes that occur in the specific CNS structures, while the neurodegeneration affects both gray and white matter. These CNS disorders share features such as neuroinflammation, reactive gliosis, excessive glutamate accumulation, or dysregulated cellular signaling pathways. Among them, accumulating evidence has fitted Wnt signaling into the role of a significant player in the pathophysiology and prevention of cerebral ischemic injury and AD. Together with its considerable functions in neuroprotection, stimulation of neurogenesis, maintaining the BBB integrity, and modulating the expression of AD-related genes, several components of the Wnt pathway (preferentially Dkk1 and GSK3β) may contribute to the development of therapies leading to better outcomes of the CNS neuropathologies. However, these still emerging, yet promising, hypotheses need to be validated. For a summary of the participation of Wnt signaling in brain ischemia and AD, please refer to Figure 2. Importantly, it is worth mentioning that while the roles of the Wnt pathway in ameliorating the negative effects of ischemia or AD seem very attractive, one should also consider that modulating this pathway means dealing with tumor suppressor genes and proto-oncogenes, and Wnt signaling hyper-activation may thus cause an aberrant growth of cells, which leads to the development of tumors [245,246].

## 6. Future Perspectives

To date, no definitive cure exists for either brain ischemia or AD. These two neuropathologies differ in the pace of their progression; on the one hand, AD develops relatively slowly and typically spans several stages [247]; on the other hand, brain ischemia starts as an acute injury, and neuronal cells die within minutes or days after the onset of the condition [8]. For this reason, it is crucial to develop an effective treatment for brain ischemia, as the intervention has to be prompt.

Currently, ischemic patients can be treated with tissue plasminogen activator (tPA), a protein that dissolves blood clots and thus restores the blood flow in the brain regions affected by a stroke. Moreover, tPA also has a neuroprotective effect [248]; however, its administration is only effective for a short time window following the onset of ischemia, and it poses an additional risk of hemorrhagic transformation [249].

Although several mechanisms involved in the pathology of AD have already been proposed, there is still a need to piece these together, once they are all discovered. Therefore, the treatment of AD is currently limited to alleviating symptoms and slowing the progression of the disease. These also include comorbidity control, diet modification, physical activity, and cognitive stimulation. Therapeutic approaches focused on the symptoms include cholinesterase inhibitors, which lessen dyskinesia and mood swings, or NMDAR antagonists, such as memantine, with a slight beneficial effect on cognition. However, these approaches neither slow the progression of AD, nor improve memory and cognitive performance. Treatment targeting the etiology of the disease includes, among others, secretase modulators, amyloid binders, and compounds preventing the Aβ aggregation. Several experimental attempts reducing Aβ and tau levels have been transferred to clinical trials; however, these studies failed to be successful [250,251]. As mentioned before, the original hypothesis of the amyloid cascade, which considered the high levels of Aβ and tau to be the main cause of AD, has already been re-evaluated and the pathological properties of Aβ are determined rather by qualitative changes in the spectrum of Aβ peptides than quantitative increases [61]. Therefore, it seems more effective to target the therapy to Aβ oligomers [252,253,254,255]. It is mainly immunotherapy with anti-Aβ oligomers or antagonists of toxin receptors, such as sigma 2/progesterone receptor membrane component 1, that results in the blockage of Aβ oligomer binding and the prevention of synaptotoxicity [256,257]. High expectations are also placed on stem cell therapy. Stem cells have not only the ability to migrate to the site of injury and differentiate to various types of neural cells, but they also produce neurotrophic factors and other protective molecules that can activate the defensive and regenerative processes [258,259,260]. Additionally, drugs that are targeted to glial cells are also considered a promising therapeutic approach. In astrocytes, these are mainly substances that increase the expression of EAATs, and thus reduce the glutamate overstimulation in neurons. To name a few, β-lactam antibiotics, ampicillin [261], ceftriaxone [262], estrogen [263], insulin [264], and riluzole [265] all appear to be effective. Regarding the development of inflammation in AD, the effects of various anti-inflammatory agents have also been investigated. Some studies showed that long-term users of non-steroidal anti-inflammatory drugs, such as ibuprofen or tarenflurbil, have a lower risk of developing AD [266]. However, clinical studies once again refuted these results [267,268]. Moreover, some other promising inflammation-targeting drugs are etanercept, a TNFα inhibitor [269], and neflamapimod, a selective inhibitor of the α isoform of p38 MAPK [270]. In addition, azeliragon or pioglitazone, a peroxisome proliferator-activated receptor γ (PPARγ) agonist, have also been shown to be promising, but failed in the clinical trials [271]. Additionally, in AD patients, a novel treatment with the monoclonal antibody aducanumab, which targets Aβ, has been proposed; however, an intense discussion about the merits of this medicament is still ongoing [272,273,274]. Since the exact molecular mechanisms and processes in the triggering and progression of AD are still not fully explored, no other cure exists at this time. Nevertheless, the utilization of the resident neurogenic cell populations in the brain, such as NSPCs or even NG2 glia, might prove useful to replace the lost cells [122,161].

As mentioned before, the fate and survival of neural cells depend to a great extent on the activity of cellular signaling pathways. Importantly, the Wnt signaling pathway plays a crucial role in both ischemic stroke and AD (Figure 2). Furthermore, the risk of these disorders is increased in the elderly and, at the same time, Wnt signaling ceases with age [159]. Additionally, since this cellular signaling pathway is known for its neuroprotective [275], anti-inflammatory [276], and neurogenic properties [162], while it can also improve cognitive functions [237], we stress the following Wnt-signaling-linked chemical compounds that may represent possible treatments of brain ischemia and AD. The first promising results come from in vitro studies, where the treatment with Wnt3a ligand increased cell survival after the administration of several toxic agents, including Aβ, and thus protected neural cells against apoptosis [277]. A means of activating canonical Wnt signaling for therapeutic purposes may represent the utilization of small-molecule inhibitors that antagonize the interaction between the Dkk1 blocker and the LRP co-receptor. Importantly, this approach does not affect the binding of the Wnt ligands to their Wnt receptors [278]. Moreover, Dkk1 attenuation reduces apoptosis [279] and prevents the loss of synapses in an AD cell culture model [280]. Therefore, restoring canonical Wnt signaling represents a promising therapeutic tool for AD [178,225]. Additionally, the activation of the non-canonical Wnt/Ca^2+^ signaling pathway by Wnt5a led to the modulation of mitochondrial dynamics, which prevented the alterations induced by Aβ oligomers [281].

It is noteworthy that several Wnt-signaling-affecting drugs have also already been tested in ischemia and AD. One such compound is the immunosuppressant rapamycin. This compound was observed to ameliorate the AD pathology via inhibiting GSK3β and elevating the expression levels of Wnt3a [282]. In addition, a glutamate modulator riluzole, which is used in the treatment of amyotrophic lateral sclerosis, is capable of activating the Wnt pathway, and therefore ameliorating oxidative stress and neuroinflammation in AD [283]. Moreover, the combination of the non-steroidal anti-inflammatory drug ibuprofen and the cholinesterase inhibitor octyl-pyridostigmine, suppresses the activity of GSK3β and protects cells against the Aβ neurotoxicity in AD [284]. Concerning ischemic stroke, exogenous estradiol administration might prove to be beneficial, since it acts via Wnt signaling and reduces the tissue damage resulting from experimental ischemic stroke in both females and males [194]. The antidiabetic medication liragitude has also been observed to exert neuroprotective properties in both cerebral ischemia and AD, and one of its putative targets is GSK3β [285]. Interestingly, nicotine is capable of stabilizing β-catenin, which might be relevant in therapeutic approaches dealing with AD [286]. Two drugs used in the treatment of bipolar disorder, valproic acid and lithium, are widely used in experimental settings as inhibitors of GSK3β. Both compounds were also used in experiments on animal models of ischemic injury and AD. In ischemia, lithium exhibited neuroprotective effects against excitotoxicity [287]. Similarly, in models of AD, lithium played a protective role, as it prevented spatial memory deficits after the injection of Aβ fibrils into the rat hippocampus [180]. It was noteworthy that the ability of lithium to stimulate cognitive functions and neurogenesis in the subgranular zone of the hippocampus in a transgenic murine model of AD was lost in the aged transgenic mice [221], possibly due to the depletion or quiescence of NSCs in the later stages of ontogeny [161,288]. The breakdown of the BBB after ischemic stroke can also be alleviated by upregulating the Wnt signaling pathway in endothelial cells via the administration of lithium [289]. Lithium has already been used in a clinical trial as a possible post-ischemic treatment. Notably there was a significantly better improvement observed in the lithium-treated patients with cortical stroke, compared to the placebo group, and almost half of the lithium-treated patients showed an enhanced motor recovery [290]. Regarding AD, a few completed or ongoing studies on the effect of lithium or other, novel inhibitors of GSK3β on the prevention of cognitive impairments, are listed on the website of the U.S. National Library of Medicine [291]. However, it is important to note that lithium is not an exclusive activator of the canonical Wnt signaling pathway, but it also acts independently of Wnt signaling [292].

Recently, natural substances ameliorating the outcomes of neuropathologies have come to light. Among others, curcumin has been shown to induce neurogenesis and neuroprotection following brain ischemia, while it also prevents age-related aggregation of the Aβ peptide and tau protein in post-ischemic dementia and AD [293,294]. The neuroprotective effect of curcumin is based on the induction of autophagy of misfolded proteins [295], and involves the inhibition of GSK3β, which activates the Wnt/β-catenin signaling pathway. This mechanism was confirmed by its repression with the Dkk1 Wnt blocker [296].

Understanding the molecular mechanisms that are implicated in the etiology of neurodegenerative diseases may have a prospect of developing novel therapeutic approaches. Recent findings identified the Wnt pathway as an important factor in the pathophysiology of ischemic brain injury and AD; however, clinically relevant modulation of this cellular signaling pathway is still to be developed. Therefore, a greater understanding of the precise mechanisms of Wnt signaling in the onset and progression of neuropathologies may pave the way for the treatment of the diseased adult CNS.

## Data Availability

Not applicable.
